# Bone Mineral Density, TBS, and Body Composition Indexes in Ukrainian Men with Parkinson's Disease

**DOI:** 10.1155/2019/9394514

**Published:** 2019-02-07

**Authors:** Vladyslav Povoroznyuk, Maryna Bystrytska, Nataliia Grygorieva, Iryna Karaban, Nina Karasevich

**Affiliations:** ^1^SI “D. F. Chebotarev Institute of Gerontology NAMS of Ukraine”, Department of Clinical Physiology & Pathology of Locomotor Apparatus, Kyiv, Ukraine; ^2^SI “D. F. Chebotarev Institute of Gerontology NAMS of Ukraine”, Department of Clinical Physiology & Pathology of Extrapiramide Nervous System, Kyiv, Ukraine

## Abstract

**Introduction:**

Current research studies demonstrate the changes of bone mineral density (BMD) in subjects with Parkinson's disease (PD); however, data about bone quality and body composition (BC) indexes are insufficient. The aim of the study was to assess the parameters of BMD, ВС, and trabecular bone score (TBS) in PD males.

**Materials and Methods:**

We performed a cross-sectional case-control research design and examined 76 males aged 50–77 years old, who were divided into two groups: first group including men without PD (*n*=38) and the second group including subjects with PD (*n*=38). Disease duration was at least 5 years; all PD participants were at levodopa therapy. BMD of lumbar spine, femoral neck, total femur, radius, and total body and TBS *L*_l_−*L*_4_ were measured using the DXA method. Whole-body DXA measures were also used for the study of total, lean, and fat masses, skeletal muscle index (SMI), appendicular lean mass index (ALMI), and fat mass index (FMI).

**Results:**

Our study showed an increased incidence of osteoporosis and significantly lower total body BMD (respectively, 1.20 ± 0.13 and 1.26 ± 0.10 g/cm^2^, *p*=0.05), but not lumbar spine and femoral neck BMDs, and higher TBS value in PD men comparing to the control group (respectively, 1.33 ± 0.12 and 1.22 ± 0.18 un., *p*=0.005). Also, we established significantly decreased lower extremities BMD indexes, but not upper extremities, spine, and trunk BMDs in PD males. The femoral neck, proximal femur, and lower extremities BMD indexes in PD men were reliably lower at the side of predominance of clinical symptoms. Parameters of appendicular lean mass and ALMI in PD males were reliably higher, but fat mass values and FMI were lower compared to the control group in the absence of significant differences in lean mass values and SMI in weight-matched control.

**Conclusion:**

Due to low BMD values, changes in BC are present in PD males, and appropriate screening and preventive strategies should be instigated to maintain bone health in PD subjects.

## 1. Introduction

Parkinson's disease (PD) is one of the most important chronic progressive brain diseases that traditionally manifests by a combination of rigidity, tremor while rest, bradykinesia, and postural instability, as well as a wide range of nonmotor manifestations—mental, vegetative, sensory, and others [1].

Systemic osteoporosis is an important complication of PD. One of the main reasons for the high fractures' risk in men with PD is low bone mineral density (BMD) [2–4], which is a crucial parameter of bone strength; however, current literary data about BMD indexes in men with PD are controversial. Current research studies demonstrate various BMD changes in PD subjects; however, mechanisms for bone involvement in these subjects are currently unclear [5–11]. Some authors [4] found that BMD in males with PD was associated with disease duration and severity; however, others [3] denied any difference in reduced BMD when comparing 5–10 years versus 0–5 years PD subjects. Additionally, it was shown that females and males with PD had significantly lower BMD values at femoral neck compared to healthy control, whereas BMD indexes at lumbar spine were decreased only in PD women compared to subjects of control groups [5]. These studies are performed primarily on the European, American, and Asian populations, and they demonstrated some national differences; however, no study was conducted yet on Ukrainian population.

Moreover, the trabecular bone score (TBS) is another critical risk factor for osteoporotic fractures which reflects the bone architecture and predicts the fractures risk as well as FRAX independently from BMD [12–15]. However, up to date, the information about TBS indexes in subjects with PD is absent.

Current literature data also have shown that body weight loss is important in PD progression. Many studies demonstrated that low body mass index (BMI) is one of the risk factors for fractures risk; however, only few ones confirmed some changes in body composition (BC) in PD subjects [16–18]. The results of these research studies are controversial; possibly, it is connected with different PD population and needs the future studies.

Therefore, the aim of the study was to assess the parameters of bone mineral density, trabecular bone score, and body composition parameters in males with Parkinson's disease. We analyzed some BMD features in men with PD depending on parts of skeleton (trunk of extremities) and predominance of clinical symptoms. Moreover, we studied the relationships between BMD and changes of fat and lean mass indexes. Also, we hypothesized the importance of TBS and BC changes in men with advanced stage of PD for bone health.

## 2. Materials and Methods

### 2.1. Study Population

We performed a cross-sectional case-control research design. The study was conducted in the Department of Clinical Physiology and Pathology of Locomotor Apparatus with collaboration of Clinical Physiology and Pathology of Extrapyramidal Nervous System Department of D. F. Chebotarev Institute of Gerontology, NAMS, Ukraine. The present research was approved by the Ethics Committee of the Institute (19/12/2014). All subjects signed the informed consent for participation in the study and treatment in the Institute clinic.

We examined 76 males aged 50–74 years old, who were divided into two groups: first group including men without PD and any other illnesses and conditions which can have the influence on bone state and turnover (control group, *n*=38) and the second group including men with PD (*n*=38). This case-control study presupposed a set of weight-matched subjects to exclude the influence of low mass on BMD parameters since it is known that weight loss is one of the symptoms in PD patients.

The diagnosis of PD was established according to the criteria of the Brain Bank of the British Society of Parkinson's Disease by a movement disorder specialist. Clinical features of PD were evaluated according to the Unified Parkinson's Disease Rating Scale (UPDRS) [19]; PD stages were assessed by Hoehn and Yahr (H&Y) classification [20]. Disease severity was categorized into moderate (H&Y stage 2, *n*=12) and severe (H&Y stage 3, *n*=26) stages. We excluded the patients with 4-5 stages of PD according to the H&Y classification, pronounced tremor, and other severe motor disturbances that interfere with conducting and evaluating of dual-energy X-ray absorptiometry (DXA) and interpretation of study results.

Mean parameters of the Unified Parkinson's Disease Rating Scale (UPDRS) [19] in PD subjects consisted of UPDRS I (mentation, behavior, and mood) subscale: 2.03 ± 1.71 un., UPDRS II (activities of daily living) subscale: 13.53 ± 4.55 un., UPDRS III (motor) subscale: 36.37 ± 10.70 un., and total count: 51.93 ± 14.79 un.

The age of PD onset in Group II consisted of 57.8 ± 8.8 years, with mean duration of PD–7.4 ± 4.2 years, respectively. All participants with PD were at levodopa therapy (mean duration: 4.75 ± 3.32 years), and its current dose consisted of 440 ± 207 mg/d.

### 2.2. Assessments

Bone mineral density of lumbar spine, femoral neck, total femur, radius, and total body and *T*- and *Z*-scores (which reflects the comparison with healthy young (20 years) adults and age-matched population, accordingly) were measured using the dual-energy X-ray absorptiometry (DXA) method (Prodigy, GEHC Lunar, Madison, WI, USA). Trabecular bone score (*L*_l_−*L*_4_) values were assessed in posterior-anterior spine by TBS iNsight® software package installed on our DXA machine (Med-Imaps, Pessac, France).

Interpretation of DXA results for men aged 50 years and older was conducted (according to the International Society for Clinical Densitometry recommendations [21]) according to the lowest *T*-score at lumbar spine, proximal femur, or femoral neck (normal bone (*T*-score > −1.0 SD), osteopenia ((≤−1.0 SD) *T*-score ˃ (−2.5 SD)), and osteoporosis (*T*-score ≤ −2.5 SD)). Moreover, we analyzed the fracture frequency (%) in groups using a questionnaire, which had the information about date and localization of all previous fractures.

We performed the anthropometric measures (height and weight), and BMI was calculated. Additionally, the lean and fat masses with calculations of skeletal muscle (lean) index (skeletal muscle mass/(height)^2^, SMI, kg/m^2^), appendicular skeletal muscle mass index (skeletal muscle (lean) mass of the limbs/(height)^2^, ALMI, kg/m^2^), and fat mass index (fat mass/(height)^2^, FMI, kg/m^2^) were measured by DXA [22, 23].

The statistical analysis was conducted by the methods of descriptive statistics. The distribution of all variables was tested using the Kolmogorov–Smirnov test. Intergroup comparisons were made using Student's *t*-test for independent variables. All parameters are represented at mean (*M*) ± standard deviation (SD). To assess the correlations between parametric variables, Pearson's correlation analysis was used. Differences in the distribution of samples were evaluated using criterion *χ*_c_^2^ test. Software package of “Statistica 10.0” Copyright© StatSoft, Inc. 1984–2011 was used during the analyses.

## 3. Results

### 3.1. Participants

We have studied BMD, TBS, and BC indexes in 76 males aged 50- to 77-years-old, who were divided into groups concerning PD presence. Both groups of subjects did not differ significantly in parameters of age, height, and weight; however, BMI in males with PD was significantly lower (*p*=0.01) than similar index in the control group. The characteristics of participants are presented in Table 1.

### 3.2. Bone Mineral Density and Fracture Frequency in Subjects with Parkinson's Disease

**Figure 1:**
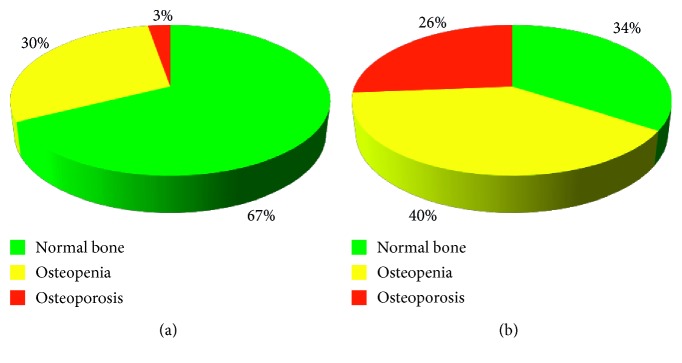
Proportion of participants from the control group (a) and men with PD (b) with different bone mineral densities (normal, osteopenia, and osteoporosis).

Low BMD (osteoporosis and osteopenia) in males with PD was registered more frequently than in control subjects (*χ*_c_^2^ = 8.2, confidential interval (CI): 7.1–39.4; *p* < 0.05 and *χ*_c_^2^ = 8.3, CI: 10.8–51.6; *p* < 0.05, respectively). Analysis of distribution in men with PD according to the frequency of bone deterioration (osteoporosis and osteopenia) demonstrated that 26% of males had osteoporosis, 40% had osteopenia, and 34% had normal BMD in comparison with 3, 30, and 67%, respectively, in the control group (Figure 1).

The analysis of fracture frequency in males depending on PD presence showed that 2.6% (*n*=1) of subjects from Group I and 21.0% (*n*=8) from Group II had low-energy fractures. Two males with PD had fracture of distal forearm, two men had hip fractures, and four men had other nonvertebral fractures. In the control group, only one nonvertebral fracture was established.

The results of our study showed that the BMD indexes in men with PD were significantly lower than similar ones in the control group only at total radius and total body; we did not find any reliable differences in BMD parameters at lumbar spine (Table 2). In addition, we have established the reliable differences of femoral neck *Т*-score between groups; however, BMD and *Z*-score had only tendency to differ (*p*=0.06 and *p*=0.11, respectively).

The analysis of BMD indexes in different parts of the skeleton found significantly lower parameters only at lower extremities (1.46 ± 0.15 and 1.38 ± 0.17 g/cm^2^; *t* = 2.13; *p*=0.04) with no significant differences at the upper extremities (1.01 ± 0.11 and 0.98 ± 0.13 g/cm^2^; *t* = 0.99; *p*=0.32), spine (1.20 ± 0.19 and 1.17 ± 0.25 g/cm^2^; *t* = 0.70; *p*=0.49), and trunk (1.01 ± 0.10 and 0.96 ± 0.14 g/cm^2^; *t* = 1.71; *p*=0.09).

Additionally, we studied BMD indexes in men with PD depending on the side of clinical symptoms' predominance compared to the other side. The significant differences in BMD at femoral neck, proximal femur, and lower extremities were obtained, where all these indexes were lower on the side with predominance of clinical symptoms (Table 3). In contrast to the above results, we did not find any reliable difference in BMD between the different parts of body (left and right) in the males of control group. The unreliable differences were various, and they were directed in opposite ways in contrast to those in subjects with PD.

Correlation analysis did not show the significant relationship between age and BMD of lumbar spine, femoral neck, and total body indexes (except for total radius, *r* = 0.56; *p*=0.0002) in males with PD in contrast to the control group where we revealed the reliable correlation between age and femoral neck BMD (*r* = 0.39; *p*=0.04), total radius BMD (*r* = 0.46; *p*=0.02), and total body BMD (*r* = 0.41; *p*=0.04). Additionally, we did not establish reliable relationship between height and different BMD indexes; however, we found this moderate or strong correlation between weight (respectively, for *L*_1_−*L*_4_: *r* = 0.73; *p*=0.0000001; femoral neck: *r* = 0.58; *P*=0.0002; total radius: *r* = 0.40; *P*=0.013; total body: *r* = 0.72; *p*=0.0000001), BMI, and parameters of BMD at various sites of skeleton.

### 3.3. Trabecular Bone Score in Subjects with Parkinson's Disease

Analysis of study parameters revealed the significant differences in another bone parameter and reflected its quality, TBS, which was reliably higher in males with PD in comparison with data of the control group (1.33 ± 0.12 and 1.22 ± 0.18 un., *t* = 2.94; *P*=0.005). However, we did not establish reliable correlation of TBS neither with age nor with BMD parameters and BC indexes in PD males; however, we revealed the significantly moderate correlation between TBS and body weight in males of the control group (*r* = 0.48; *P*=0.03).

### 3.4. Body Composition in Men with Parkinson's Disease

Analysis of body structure in subjects depending on PD presence showed the significantly lower fat mass value in males with PD in comparison to the control group (18.86 ± 8.67 and 23.94 ± 7.91 kg, respectively, *t* = 2.59; *P*=0.01). In addition, we established the reliably lower parameters of FMI in men with PD (6.04 ± 2.67 and 7.86 ± 2.41 kg/m^2^, respectively, *t* = 3.04; *P*=0.003). However, we did not find any reliable differences of lean mass value (55.72 ± 6.97 and 57.59 ± 5.56 kg, respectively, in Groups I and II; *t* = 1.26; *p*=0.21) and SMI (18.38 ± 1.82 and 18.57 ± 1.53 g/m^2^; *t* = 0.49; *p*=0.63) depending on PD presence.

In contrast to these data, the index of appendicular lean mass was significantly higher in men with PD compared to the parameter of the control group (25.65 ± 2.73 and 23.44 ± 3.29; *t* = 3.09; *p*=0.003). We also found higher ALMI in males with PD (8.27 ± 0.74 and 7.74 ± 0.96 g/m^2^; *t* = 2.63; *p*=0.01 respectively, in Groups I and II).

**Figure 2:**
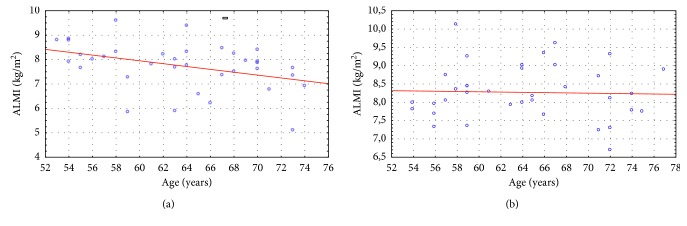
Correlations between age and ALMI in males of the control group and subjects with PD. (a) Group I. (b) Group II.

In addition, we established the reliable correlations (Figure 2) between age and lean mass (*r* = −0.61; *p*=0.00009), SMI (*r* = − 0.36; *p*=0.03), appendicular lean mass (*r* = −0.64; *p*=0.00003), and ALMI (*r* = − 0.39; *p*=0.02) in the absence of the significant correlations of these indexes in the subjects with PD. Also, we did not find any reliable correlations between age and fat mass and FMI in both groups.

Analysis of relationship between BMD of various sites and parameters of BC showed significant correlations between femoral neck BMD and total fat and FMI in both groups (FMI: Group I–*r* = 0.38; *p*=0.03; Group II–*r* = 0.42; *p*=0.01, respectively). The same relationships were established for lumbar spine BMD (FMI: Group I–*r* = 0.41; *p*=0.01; Group II–*r* = 0.22; *p*=0.001, respectively) and total body BMD (FMI: Group I–*r* = 0.35; *p*=0.04; Group II–*r* = 0.45; *p*=0.006, respectively).

Moreover, parameters of SMI showed slight yet reliable correlation with total body BMD in PD subjects (*r* = 0.39; *p*=0.02), whereas relationships at lumbar spine and femoral neck were insignificant. Additionally, reliable correlations between SMI and BMD in men with PD were established at lower extremities (*r* = 0.39; *p*=0.02) but not at upper extremities (*r* = 0.19; *p*=0.28). In contrast, slight yet significant correlations between SMI and lumbar spine BMD (*r* = 0.35; *p*=0.04), femoral neck BMD (*r* = 0.37; *p*=0.03), and total body BMD (*r* = 0.47; *p*=0.004) were found in the control group.

**Figure 3:**
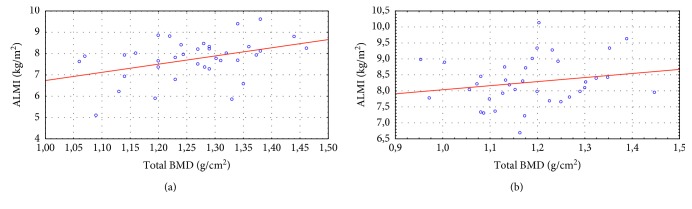
Correlations between total body BMD and ALMI in subjects of control group and males with PD. (a) Group I. (b) Group II.

The parameter of ALMI demonstrated the reliable moderate correlation with total body BMD in the control group (*r* = 0.4; *p*=0.02), but not in males with PD (*r* = 0.20; *p*=0.25) (Figure 3).

## 4. Discussion

Osteoporosis is a major nonmotor complication of PD [6–8, 19]. Recent review demonstrated that up to 61% of males with PD and 91% of women have osteoporosis [8]. In addition, current evidences suggest that PD subjects are at increased risk of osteoporotic fractures [8, 24]; however, exact mechanism of bone loss in these patients is unclear. Literature reviews confirm the important role of different intrinsic (disease-related) and extrinsic (environmental) factors in accelerated bone loss and osteoporosis development. Low BMD and BMI, vitamin D deficiency, immobilization, high risk of falling, some endocrine and nutritional factors, and elevated homocysteine levels due to high-dose levodopa treatment are some of them [6–8, 24, 25].

Current data about BMD in PD subjects are controversial and depend on sex, disease duration, and severity. In Ukraine, our study was the first one, and it confirmed the high frequency of low BMD among men with PD (60%) and high risk of fractures (21%), which is similar to the results of other studies, in particular by Turkish and British researchers [4, 11]. In addition, our study demonstrated significantly lower BMD parameters of distal radius and total body in men with PD than similar in the control group, and the difference in femoral neck BMD reached 7%, at total body–5%.

In contrast to other research studies, our results did not confirm the significant differences of lumbar spine BMD and marginal confidence values at femoral neck BMD between the control group and subjects with PD, which can be related to the stage of PD (2-3 according to H&Y), as well as some other factors. Moreover, analysis of BMD indexes in different parts of the skeleton found significantly lower parameters only at lower extremities but not at upper extremities, spine, and trunk, which can confirm that bone loss in these subjects may have been started from the lower part of skeleton. In addition, analysis of BMD indexes in men with PD depending on predominance of clinical symptoms on body side established the reliable differences at femoral neck, proximal femur, and lower extremities, where all these indexes were lower at side with predominance of clinical symptoms. Also, we did not find any significant correlation between BMD indexes and age (except for total radius) in males with PD that is consistent with data of other researchers [3].

Nowadays, measuring BMD using DXA is the gold standard for diagnosing osteoporosis; however, it does not directly reflect deteriorations of bone structure. TBS is another DXA parameter that correlates with 3D parameters of bone microarchitecture. Today, combining the TBS with BMD improves fracture prediction in women and men. Recent literature data showed that reduced TBS was associated with prior major osteoporotic fracture, glucocorticoid use, and various diseases (rheumatoid arthritis, chronic obstructive pulmonary disease, diabetes mellitus, and so on) and strongly associated with many risk factors of osteoporotic fractures [14, 15]. In contrast to other research studies, our study established the significantly higher TBS in males with PD in comparison with data of the control group, paralleling with absence of reliable differences in lumbar spine BMD that requires further study depending on the stage of the disease, tremor rate, concomitant therapy etc.

According to the existing data, low BMI is another substantial factor which influences BMD and fracture risk; however, up to date, little is known about BC indexes in PD males. Some studies demonstrated the increase rate of sarcopenia in subjects with the advanced stage of PD [18, 26–28].

According to the study results of Revilla et al. [17], the index of fat mass was significantly higher (*p* < 0.01) and parameters of the lean body mass and water content were lower (*p* < 0.001 for each) in PD men, but not in women in comparison with healthy control. Another study, that was performed by Sakajiri and Takamori [16] in males and females with PD, showed that the reductions of body weight and BMI are significantly higher in females with PD than in males and there are not any reliable correlation between the disease duration and changes in body weight and BMD in contrast to significant negative correlation with ratio of body fat.

Contrary to the data above, some current study by Wilczyński and Półrola [27] did not confirm BC changes in PD subjects but demonstrated the sex features in fat mass (%), fat-free and muscle mass (kg), visceral fat, and other parameters of BC. Despite the fact that some studies confirmed the presence of sarcopenic obesity in PD subjects, some recent research studies demonstrated that appendicular lean body mass reliably correlated with cerebral cholinergic innervations in PD subjects independent of age [28].

Considering the meaningful influence of low BMI on BMD parameters and their significant role in disease course, the design of our study presupposed the selection of a weight-matched control. Despite this option, our study found the significantly lower fat mass values and FMI in PD men in comparison with the control group. However, we did not find any reliable differences of lean mass values and SMI. Contrary to other studies, the parameters of appendicular lean mass and ALMI were significantly higher in PD males compared with parameters of the control group that requires future studies in subjects with PD depending on sex, disease severity, duration, etc.

Analysis of relationships between BMD of various sites and parameters of BC showed significant correlations between femoral neck BMD and total fat and FMI in both groups. The same relationships were established for lumbar spine BMD and total body BMD. ALMI demonstrated the reliable correlation with total body BMD in the control group, but not in males with PD. Moreover, parameters of SMI showed reliable correlation with total body BMD, but not lumbar spine and femoral neck BMD in PD subjects. Interestingly, reliable correlation between SMI and BMD in men with PD was established at lower extremities, but not at upper extremities. In contrast to the data above, we established the significant correlations between SMI and lumbar spine, femoral neck, and total body BMD indexes in the control group. Interestingly, these findings were present in the absence of significant differences in weight parameters in PD males. These results can be related with PD stage and sex particularities and also require further research.

The limitations of current research include cross-sectional design, sample size, and inclusion of only men population with 2^d^-3^d^ stage of disease (according to the H&Y) that did not allow determining the effect of some other PD parameters on bone tissue state. Further large-scale longitudinal studies are required to find the association between PD and osteoporosis more fully for the determination of bone-safe strategy for subjects with PD.

## 5. Conclusion

The results of our study showed an increased incidence of osteoporosis and significantly lower total body BMD, but not lumbar spine and femoral neck BMD indexes in men with PD compared to the control group. We established significantly decreased parameters of lower extremities BMD, but not upper extremities BMD, spine BMD, and trunk BMD in PD males. In addition, femoral neck, proximal femur, and lower extremities BMD indexes in subjects with PD were reliably lower at side with predominance of clinical symptoms.

Also, we revealed the reliably higher parameter of appendicular lean mass and ALMI and lower fat mass value and FMI in PD males compared to the control group in absence of significant differences in lean mass index and SMI in weight-matched males.

Due to low BMD values, changes in BC are present in PD males, and appropriate screening and preventive strategies should be instigated to maintain bone health in PD subjects. Complex and regular assessment of skeletal integrity levels is essential for disease progression and prevention of such vital complications as low-energy fractures.

## Figures and Tables

**Table 1:** Characteristics of participants.

Index/group	Group I	Group II	*t*	*p*
Age (years)	62.97 ± 6.83	64.37 ± 6.61	0.90	0.37
Height (m)	1.74 ± 0.06	1.76 ± 0.06	1.60	0.11
Weight (kg)	84.11 ± 11.92	80.11 ± 11.37	1.50	0.14
Body mass index (kg/m^2^)	27.78 ± 3.36	25.81 ± 3.03	2.68	0.01

*Note*. Data are presented as mean ± SD.

**Table 2:** Bone mineral density indexes in males depending on PD presence.

Index/group	Group I	Group II	*t*	*p*
*L*_1_−*L*_4_				
BMD (g/cm^2^)	1.31 ± 0.23	1.24 ± 0.32	1.07	0.29
*Т*-score (SD)	0.73 ± 1.88	0.17 ± 2.67	1.05	0.30
*Z*-score (SD)	0.99 ± 1.68	0.55 ± 2.42	0.92	0.36

Femoral neck				
BMD (g/cm^2^)	0.97 ± 0.13	0.91 ± 0.15	1.90	0.06
*Т*-score (SD)	−0.75 ± 0.95	−1.22 ± 1.13	1.96	0.05
*Z*-score (SD)	0.16 ± 0.88	−0.19 ± 0.97	1.60	0.11

Total radius				
BMD (g/cm^2^)	0.77 ± 0.08	0.71 ± 0.11	2.37	0.02
*Т*-score (SD)	0.14 ± 1.14	−0.59 ± 1.66	2.07	0.04
*Z*-score (SD)	0.60 ± 1.09	0.00 ± 1.53	1.81	0.07

Total body				
BMD (g/cm^2^)	1.26 ± 0.10	1.20 ± 0.13	2.02	0.05
*Т*-score (SD)	0.49 ± 1.23	−0.24 ± 1.64	2.17	0.03
*Z*-score (SD)	0.55 ± 1.13	0.00 ± 1.30	1.97	0.05

*Note*. Data presented as mean ± SD.

**Table 3:** Bone mineral density indexes in males with PD according to the predominance of clinical symptoms (g/cm^2^).

Index/group	A side with a predominance of clinical symptoms	The opposite side	*p*
Femoral neck	0.92 ± 0.15	0.94 ± 0.18	<0.05
Proximal femur	1.03 ± 0.17	1.06 ± 0.19	<0.05
Upper limbs	0.98 ± 0.14	0.99 ± 0.13	>0.05
Lower limbs	1.38 ± 0.17	1.40 ± 0.18	<0.05
Total body	1.20 ± 0.13	1.21 ± 0.12	>0.05

## Data Availability

All data used to support the results of this study are stored by and available from the corresponding author upon request.
